# Clinical applications and utility of ctDNA in cervical cancer and its precursor lesions: from screening to predictive biomarker

**DOI:** 10.1186/s12935-023-03132-0

**Published:** 2023-12-18

**Authors:** Li Li, Yixin Tong, Jianhong Wu, Xiangshang Xu

**Affiliations:** 1 Cancer Biology Research Center (Key Laboratory of the Ministry of Education), Tongji Hospital, Huazhong University of Science and Technology, Wuhan, China; 2 GI Cancer Research Institute, Tongji Hospital, Huazhong University of Science and Technology, 1095 Jiefang Avenue, Wuhan, China

**Keywords:** Cervical cancer, ctDNA, HPV, Biomarker

## Abstract

Cervical cancer is a leading cause of gynecological cancer death in the world. Human papillomavirus (HPV) is the most causative factor of cervical cancer. In addition, many genetic factors are involved in cervical cancer development. Most studies focus on cervical samples to do research work about cervical cancer and precancerous lesions, but no sensitive or specific biomarkers were found. High-throughput genomic technologies are able to capture information from tumors and precancerous lesions in blood, thus providing a new way for the early diagnosis of cervical precancer and cervical cancer. Blood is an ideal specimen for detecting cancer biomarkers because it contains a lot of information, such as circulating tumor cells and circulating tumor DNA (ctDNA). This article reviews the clinical use and challenges of blood ctDNA testing in patients with cervical precancer and cervical cancer.

## Introduction

In November 2020, the Director-General of the World Health Organization (WHO) called on the world to speed up eliminating cervical cancer, which includes the following targets for 2030 in each of the three pillars: 90% coverage of eligible girls with human papillomavirus (HPV) vaccination, 70% coverage of high-performance testing screening and 90% of women who have positive screening tests or cervical lesions are appropriately treated [1]. HPV vaccine and screening, as the primary and secondary prevention measures, have significantly reduced morbidity and mortality in high-income countries. Nevertheless, among women in low-income countries, cervical cancer remains the most commonly diagnosed cancer and the main cause of cancer death. Most of these countries are in sub-Saharan Africa, Melanesia, South America and Southeast Asia [2]. Cervical cancer is still an important public health problem worldwide. There were an estimated 604,000 new cases and 342,000 deaths in 2020 [3].

The burden of cervical cancer can be effectively reduced through affordable and accessible screening programs and effective vaccination programs. Since the first HPV vaccine was approved 16 years ago (Table 1), the incidence and prevalence of HPV infection and cervical lesions have decreased in areas covered by the HPV vaccine [4]. When using the three-dose plan for young women (< 25 years old) with negative HPV, the efficacy of the HPV vaccine is close to 100%, which can prevent persistent infection and precancerous lesions related to the HPV vaccine type [5]. There are many obstacles to the implementation of vaccines, including high cost, unavailability, and lack of proper storage or transportation conditions. The main challenge is that the vaccines do not protect all types of HPV.

**Table 1:** Current licensed human papillomavirus vaccines

Brand Name	Gardasil	Cervarix	Gardasil 9	Cecoline
**Manufacturer**	Merck & Co	GlaxoSmithKline	Merck & Co	Xiamen Innovax
**Valency**	quadrivalent	bivalent	nonavalent	bivalent
**VLP Types**	HPV6/11/16/18	HPV16/18	HPV6/11/16/18/31/33/45/52/58	HPV16/18
**First Licensed**	2006	2007	2014	2021
**Doses Recommended**	2 or 3 doses, depending on age at initiation	2 or 3 doses, depending on age at initiation	2 or 3 doses, depending on age at initiation	2 or 3 doses, depending on age at initiation
**Administration**	Intramuscular	Intramuscular	Intramuscular	Intramuscular
Expression System	*Saccharomyces cerevisiae* (Baker’s yeast)	*Trichoplusia ni* Rix4446 cell using the *baculovirus* expression vector	*Saccharomyces cerevisiae* (Baker’s yeast)	*Escherichia coli*

Cervical cancer screening is an effective intervention to prevent advanced disease and death of cervical cancer (Table 2). The WHO indicates that long-term and systematic screening can reduce cervical cancer death by 93% [1]. Compared with traditional cytological methods, HPV detection is a more effective, reliable and adaptive screening method [6]. However, high-risk HPV (HR-HPV) DNA testing can also detect transient infection without clinical significance. It leads to poor specificity in detecting cervical intraepithelial neoplasia (CIN), especially in young women [7]. Finding a highly sensitive and specific genetic and molecular marker for early diagnosis of cervical precancerous lesions and tumor progression is an important task of gynecological oncology.

**Table 2:** Current approaches to cervical cancer screening. WHO recommends using HPV DNA detection as the primary screening test among the general population of women, and using partial genotyping, colposcopy, visual inspection with acetic acid or cytology to triage women after a positive HPV DNA. HPV mRNA is an alternative method to HPV DNA tests for HPV detection

Molecular tests	Cytology tests	Visual inspection
HR-HPV DNA	Pap smear	Visual inspection with acetic acid or with Lugol’s iodine mainly by the colposcope
HR-HPV mRNA	Liquid-based cytology (LBC)	
	Dual staining to identify *p16* and *Ki-67*	

Serum biomarkers play an important role in monitoring many malignant neoplasms. Cervical squamous cell carcinoma is the predominant histological type, while about 20% of cervical cancers are adenocarcinomas [8]. Squamous cell carcinoma antigen (SCC-Ag) is a reliable squamous cell carcinoma biomarker. SCC-Ag levels may vary depending on the tumor stage, lymph node status, and clinical outcome [9]. However, only 64% of squamous cell carcinoma patients and 25% of adenocarcinoma patients have increased SCC-Ag levels. In contrast, only 42.6% of squamous cell cervical cancer patients and 18.9% of adenocarcinoma patients had increased levels of CA-125 [10].

Cell-free DNA (cfDNA) means the extracellular DNA molecules found in peripheral blood or body fluids from any type of cell [11]. CfDNA is usually called circulating tumor DNA (ctDNA) when released from tumors or circulating tumor cells. Over the past decade, researchers have given great attention to ctDNA because of its great potential as a minimally invasive biomarker in the carcinogenic process. It reflects tumor load according to ctDNA concentration, gene methylation and chromosome changes [12]. An ideal blood-based approach for screening and triaging precancerous lesions and cervical cancer requires high sensitivity and specificity.

Additionally, it should be easy to sample and inexpensive to conduct population-based screening. The blood ctDNA test is expected to meet this requirement. This review summarizes and discusses the application and triage performance of ctDNA in cervical precancerous lesions and cervical cancer.

### Etiological factors of Cervical Cancer

More than 90% of cervical cancer tissues are infected by high-risk HPV, which is the leading cause of cervical cancer [13]. More than 200 HPV types are characterized, and about 40 HPV types can infect anogenital mucosa [14]. According to their ability to induce cervical cancer, they can be divided into two categories: high-risk HPV (HR-HPV) and low-risk HPV (LR-HPV) [15]. HR-HPV family members are associated with high-grade lesions and invasive cervical cancer. And they are further divided into HR-HPVs, probable HR-HPVs and possible HR-HPVs [16]. On the other hand, LR-HPV types are mainly related to genital warts and benign cervical lesions [15].

According to the International Agency for Research on Cancer (IARC), 12 types of HPV (HPV16, 18, 31, 33, 35, 39, 45, 51, 52, 56, 58 and 59) have now been uniformly listed as HR-HPV (the first group of human carcinogens) [17]. HPV68 has been listed as a possible HR-HPV (also known as IARC Group 2 A), and the other seven types are listed as possible HR-HPV (HPV26, 53, 66, 67, 70, 73, and 82; also known as IARC Group 2B). HPV16 and HPV18 cause approximately 70% of HPV-related cervical cancer. They are the two most common HPV types [18]. There are three physical regions in the viral genome: the early (E) gene regions, the late (L) gene regions, and the long control region (LCR) [19]. The early regions of the HPV genome include seven proteins: E1, E2, E1 ^ E4, E5, E6, E7, and E8 ^ E2. E1 protein and E2 protein are responsible for modulating the transcription and replication of HPV viral DNA. E5, E6 and E7 are the oncogenes of the virus that induce immortalization and transformation of cells [20]. Although the role of viruses E5 in carcinogenesis has not been studied too clearly, E6 and E7 block cell checkpoints by acting on carcinogenic and tumor suppressor proteins and miRNAs [20]. Primary and secondary capsid proteins are encoded by the late region: L1 and L2 [19].

### Detection platform of ctDNA

#### PCR-based assays

PCR-based methods have acceptable sensitivity and high specificity for a limited number of known sequences or mutations. These platforms include sensitive technologies, such as quantitative PCR (qPCR), droplet digital PCR (ddPCR), and amplification refractory mutation system (ARMS) [21]. Early studies used the qPCR method, which showed relatively high specificity but low sensitivity. When using qPCR, only 12–25% of patients with cervical cancer have HPV DNA in their serum. Therefore, it is challenging to use qPCR and detect a small amount of ctDNA in blood [22].

In the 1990s, digital PCR (dPCR) was used to measure mixtures of single nucleotide variants with better analytical sensitivity [23]. dPCR has been further developed through the development of BEAMing (beads, emulsions, amplification and magnetism), and its potential has been emphasized in some of the earliest cancer ctDNA measurements [24]. Today, ddPCR or array-based dPCR can perform thousands of reactions simultaneously. ddPCR is more sensitive and repeatable than qPCR, and can be used for absolute quantification of ctDNA in samples [25]. Many ctDNA studies use ddPCR to detect rare events because ddPCR is simpler and cheaper than most sequencing-based alternatives. In ddPCR, DNA samples are divided into thousands of droplets prior to PCR amplification and then randomly distributed. Commonly, there is zero or one target, but sometimes several targets will be in one droplet. The target was then amplified by PCR in a single droplet. A fluorescent probe is used to detect whether the droplet contains the target sequence. In mutation-based ctDNA detection, a single PCR reaction is usually performed by amplifying wild-type and mutant templates, and two probes with different markers are used to distinguish [26]. A meta-analysis analyzed 10 studies that explored whether HPV ctDNA can be a reliable biomarker of cervical cancer. It showed that two studies using ddPCR for detection reported higher sensitivity (83%,90%) and specificity (100%,100%) compared with those using qPCR [27].

To some extent, PCR-based multiple mutation analysis is possible, but DNA needs to be subdivided into different detection items. This can be achieved when there is a large amount of DNA, but in the case of limited DNA, such as when analyzing ctDNA, it will lead to a loss of sampling noise and sensitivity because rare mutant molecules will be omitted [28].

#### Next generation sequencing (NGS)-based assays

NGS-based assays, such as tagged-amplicon deep sequencing (TAM-Seq) and personalized cancer profiling by deep sequencing (CAPP-Seq), are designed to detect multiple types of mutations or sequences, including indentation, rearrangement and copy number changes, and they also show high sensitivity and specificity [29]. In general, this candidate-based strategy requires prior knowledge of the tumor genome and can detect mutations or sequences with a highly specific allele frequency of 0.01%. For HPV in cervical cancer patients, the detection threshold of the NGS method is 10 times lower than that of digital PCR, and the sensitivity and specificity are 100% [30]. Forshew et al. invented TAm-Seq which could query six genes in the large genome region that span 5995 bases to detect the low-frequency mutations in cfDNA [31]. In 2018, Gale D. et al. developed the InVision™ liquid biopsy platform to identify clinically relevant somatic changes in ctDNA with a low frequency of 35 cancer-associated genes. This amplicon-based next-generation sequencing utilizes enhanced Tam-Seq™ (eTAm-Seq™) technology [28]. Whole exome sequencing has also appeared. The cost is much lower because it only involves the sequencing of exons. Using NGS to analyze ctDNA is still expensive and time-consuming, and its value in clinical practice is limited.

There is no repeat sequence in the HPV genome suitable for more sensitive qPCR/dPCR detection. In addition, the sequence of HPV genotypes varies greatly among high-risk carcinogens. These features make the HPV assay ideal for hybridization capture sequencing because of its flexibility to integrate hundreds of decoys across the entire genome of multiple viral genotypes [32]. At the same time, HPV-seq could quantitatively detect ctDNA fragments, which include HPV genotype, HPV genome localization and ctDNA fragment length distribution, which is not easy to infer by qPCR/dPCR [32]. Because of these shortcomings, researchers have proposed new technologies that have significantly improved assay speed, performance and sample size. For example, multiplex PCR combined with next-generation sequencing can greatly reduce background noise [33].

### Clinical application of ctDNA in Cervical cancer

#### HPV circulating Tumor DNA (HPV ctDNA)

Almost all cervical cancer is driven by cancer-causing HPV. The virus genome can be used to distinguish ctDNA from cfDNA that comes from other cellular sources. Cancer associated with HPV may contain integrated HPV DNA, extrachromosomal virus DNA, or a mixture of both. Integration usually destroys the viral *E1* gene and the *E2* gene, thereby alleviating transcriptional repression of the carcinogens *E6* and *E7* [34]. In tumors with full extrachromosomal viral DNA, the viral genome usually obtains genetic or epigenetic changes, resulting in the imbalance of *E6/E7* gene expression [35]. Detecting and quantifying HPV DNA in blood (Table 3) can be used to optimize cancer management. Its inherent application fields include early diagnosis and pre-treatment evaluation, efficacy monitoring and post-treatment monitoring.

**Table 3:** Studies measuring circulating HPV DNA in cervical cancer

Markers for detection	Detection method	Detection rate(%)	Number of patients	Reference
*L1/E7*	qPCR	25.47	106	12
*vh-DNA/E7*	qPCR	68.75/81.25	16	26
*vh-DNA*	Semi-nested PCR	23	21	27
*E7/vh-DNA*	ddPCR	63/39	94/23	28
*L1/E6/E7*	PCR enzyme immunoassay	45	94	29
*E7*	qPCR	18.2	55	30
*L1/E7*	ddPCR	58.5/55.8	118/138	31
*E7*	ddPCR	100	19	32
*L1*	qPCR	14.55	55	48
*E7*	qPCR	48.48	33	49
*E6*	nested PCR	24.4	45	50
*E6/ E7*	qPCR	94.28	140	53
*E6/ E7*	nested PCR	20	20	56
*L1*	nested PCR	65	17	57
*E7*	E7-MPG/ ddPCR	77.63/64.47	76/76	54
HPV DNA	NGS-based CaptHPV	96.77	62	55

#### Circulating virus-host chimera DNA (vh-DNA)

The relationship between HPV DNA integration and carcinogenesis is reflected in the increase in integration-positive cells during the histological progression from precancerous lesions to invasive carcinoma. HPV integration follows two patterns: direct integration of incomplete individual viral genomes and circular integration of multiple genomes [36]. In the process of tumor turnover, HPV DNA integrated into the human genome is segmented and released, resulting in the detection of tumor-specific *vh-DNA* as ctDNA. An early qPCR study examined HPV human junction sequence and HPV DNA in blood samples [37]. This study showed that HPV-human junction sequences and HPV DNA can be detected in 85% and 100% of stage II-IV cancer patients [37]. Their levels in the blood decrease due to treatment and increase when they recur.

The difference in detecting the two fragments may be low HPV insertion load or DNA derived from episomal molecules. Viral-cellular junction sequence has high tumor specificity. Katrin Carow et al. studied the clonality of cervical cancer in the background of intratumoral heterogeneity and analyzed whether they were suitable for detecting circulating tumor DNA in the serum of cervical cancer patients [38]. The integration points in 7 of the 8 tumors were evenly distributed in different regions. Only one showed intratumoral heterogeneity. Among the 21 preoperative serum samples, 5 specific junction fragments were detected. The junction-based ctDNA detection was closely associated with the reduction of recurrence-free rate. Emmanuelle Jeannot et al. indicated that testing of *vh-DNA* by ddPCR was 39% lower than that of HPV *E7* in cervical cancer (70%) [39]. Because the viral integration site of each patient is unique, this marker has a high specificity, much higher than the specificity of the SCC-Ag marker.

#### Circulating HPV viral genome (HPV ctDNA)

Every full-length HPV genome (exon or linearized genome) could yield about 50 different cfDNA sequences. The median size of the HPV ctDNA sequence was 146 bp, 22 bp shorter than that of human-mapped cfDNA. [32] In an early study, serum samples from 94 patients with cervical cancer and follow-up samples from 24 patients were tested using a PCR enzyme-linked immunoassay [40]: of all samples investigated, 45% of patients had positive serum HPV DNA collected at initial diagnosis; patients who were HPV DNA positive at diagnosis but did not have recurrence after treatment had serum retesting and showed all were HPV DNA negative; 10 of 13 patients tested positive for HPV DNA (range 0-423 days) prior to clinical diagnosis of recurrence. This study suggests that serum HPV DNA appears to reflect the biological activity of the tumor.

Another early study examined DNA extracted from 112 stage IB to IIA cervical cancer patients with L1 consensus primer and 16 and 18 type-specific *E7* primer [22]. Hsu et al. found that only 24.1% had positive HPV ctDNA; Positive serum HPV DNA was significantly associated with lymphovascular infiltration and deep stromal infiltration (with or without parametrial dilatation), pelvic lymph node metastasis, large tumors, and elevated SCC-Ag levels. When serum HPV DNA was used to predict high-risk patients requiring adjuvant therapy, the sensitivity, specificity, positive predictive value, and negative predictive value were 45.2%, 88.6%, 70.4%, and 72.9 respectively [22]. Dong et al. analyzed the incidence of HPV16 and HPV18 *E7* ctDNA in the plasma of 175 patients with invasive cervical cancer and 57 patients with carcinoma in situ by routine PCR and real-time quantitative PCR [41]. When using routine PCR, they found HPV16 or HPV18 E7 DNA in 6.9% (11 of 175 cases) of invasive cervical cancer cases (18.1% were HPV16 or HPV18 positive in the genital tract) and 1.8% (1 of 57 cases) of carcinoma in situ. Quantitative PCR found the highest concentration of HPV DNA in patients with invasive cervical cancer. Cheung et al. collected blood samples from 138 patients with cervical cancer (stage I-V) before treatment, whose tumor samples expressed HPV16 or HPV18, and tested their HPV *E7* and *L1* sequences [42]. Most patients (71.7%) had HPV16, 78.3% had squamous cell carcinomas, and 82.6% had stage IB-II disease. Plasma ctDNA from 61.6% (85/138) of patients contained HPV *E7* and *L1* sequences. In a retrospective study, the HPV ctDNA of 19 metastatic HPV16 or HPV18-positive cervical cancer patients was measured using ddPCR [43]. Nine patients received immunotherapy using tumor-infiltrating lymphocytes (TILs). All 19 patients with HPV-positive metastatic cervical cancers had been detected for HPV ctDNA, while the 45 healthy blood donors had no HPV ctDNA. Of the 87 consecutive serum samples from 9 patients receiving TIL immunotherapy, 87 (100%) correctly identified the HPV genotype in the patient’s tumor. There was a transient HPV ctDNA peak 2–3 days after the infusion of TIL in three patients with objective cancer regression after TIL treatment. Based on this, HPV ctDNA is useful for detecting antitumor activity in cervical cancer patients as well as for monitoring their long-term survival.

A retrospective cohort study (n = 41) and a prospective cohort study (n = 14) by Cabel et al. [44] evaluated blood and tissue samples collected from 55 HPV-positive patients undergoing chemoradiotherapy (CRT) for locally advanced cervical cancer (LACC). Genotype-specific ddPCR successfully detected HPV ctDNA in 69% of LACC patients before CRT, including 9 patients with rare genotypes. There was a correlation between HPV ctDNA levels and HPV copy numbers in tumor samples. Detection of HPV ctDNA before CRT is related to tumor stage and lymph node status. In the prospective cohort study, the presence of HPV ctDNA was associated with lower disease-free survival (DFS) and overall survival (OS) at the end of CRT. The role of ctDNA in cervical cancer was explored in a meta-analysis [27]. Fifteen studies involving 684 patients were analyzed. They found that detecting HPV ctDNA in cervical cancer patients may be a noninvasive early dynamic tumor biomarker with high specificity and medium sensitivity. Another meta-analysis focused on HPV ctDNA and pointed out that its existence was closely related to HPV-associated cancers compared with healthy donors [45].

#### Circulating HPV viral genome methylation

Methylation of DNA results in the translocation of transcription factors, thereby altering chromatin structure and DNA topology and thus altering gene expression [46]. A virus’s genome methylation negatively or positively affects its transcription [7]. Many studies have examined methylation changes in the HPV genome to identify markers that differentiate CIN from cervical cancer. With the understanding of the molecular mechanism of HPV-mediated carcinogenesis, the association between CIN/cervical cancer and methylation at the CpG sites of HPV *L1, L2, E2-E4, E5*, and *URR* has been well studied [7]. However, few studies focus on HPV genome methylation based on blood samples.

#### Circulating aberrant cell DNA methylation

Methylation alteration is an epigenetic decoration that regulates the expression of genes without altering DNA sequences. It is critical to the progress of cervical cancer in the pathogenesis and reflects the prognosis and treatment sensitivity in clinical practice [47]. Host cell DNA can also be methylated during carcinogenesis. Aberrant DNA methylation includes hypomethylation and hypermethylation. Most genes are hypermethylated in high-grade CIN and cervical cancer; only three promoter regions (*STK31, rDNA, and COL17A1*) are hypomethylated.

The methylation status of *CDH1* and *CDH13* in 93 cervical cancer patients’ serum samples was determined by Widschwendter et al. [48]. Aberrant methylations in the 5’-region of *CDH1* or *CDH13* were found in 43% (40 of 93) patients. In univariate and multivariate analyses, patients with cervical cancer with unmethylated *CDH1/CDH13* in serum had significantly better DFS. Another study found that patients with positive serum *CDH1* methylation had 7.8 times the risk of death and 92.8 times the risk of recurrence [49]. The *MEG3* methylation level in local tissues and plasma of cervical cancer patients was significantly higher than those in adjacent normal tissues and healthy participants’ plasma [50]. The authors found that plasma *MEG3* methylation was highly discriminative in predicting HR-HPV infection and lymph node metastasis. In addition, hypermethylation of *MEG3* in plasma was related to worse recurrence-free survival and OS in patients with cervical cancer. Methylated *SIM1* was detected in 36.6% (15/41) of ctDNA samples. The consistency with the matched cancer tissues was 41.5% (17/41), with a sensitivity of 38.5% and a specificity of 100% [51]. Rong G. *et al*. found an overall recombination rate of 93.3% for *CADM1* promoter methylation status between 45 paired tissue and plasma samples [52]. Statistical differences in plasma *CADM1* methylation levels were found between cervical cancer patients with and without lymph node metastases or cervical tumor patients with and without distant metastases.

#### ctDNA mutation analysis

HPV tumorigenesis is quite complex. Although E6 and E7 are necessary conditions for initiating and maintaining transformed phenotypes, the long progression from precancerous lesions to invasive cancers shows that several other carcinogenic events are critical to malignant progression.

Like most other carcinomas, cervical cancer has its mutation profiles, *ERBB2/PI3K/AKT/mTOR* signal is the most affected [53]. The pre-treatment plasma samples of 117 Chinese women with primary invasive cervical cancer were investigated [54]. They used ddPCR to find that 22.2% of these patients had *PIK3CA* mutations in plasma, including *p.E542K* or *p.E545K*. The mutation status of *PIK3CA* was closely related to tumor size. Chromosome arm 6p is one of those most frequently involved in a loss of heterozygosity (*LOH*) in cervical carcinoma patients. 62 patients with cervical cancer received definite radiotherapy [55]. They found that *LOH* on chromosome 6p21.2 was associated with recurrence after radiotherapy, independent of the size and stage of the tumor.

Lee S. et al. designed a panel of 24 genes related to cervical cancer to test and describe the patterns of somatic single nucleotide variation, uncertainty and copy number variation [56]. They used next-generation sequencing technology to analyze the panel: In 24 cervical cancer patients, 18 of the 24 genes in the NGS cervical cancer group had mutations, including somatic changes in the mutant genes (*ZFHX3*-83%, *KMT2C*-79%, *KMT2D*-79%, *NSD1*-67%, *ATM*-38%, and *RNF213*-27%). They proved that *RNF213* mutation might be a monitoring marker of chemotherapy and radiotherapy reactions. Tian, X. et al. conducted deep sequencing analysis on 322 cancer-associated genes in plasma cfDNA. They matched leukocytes in 173 consecutive blood samples from 82 patients with LACC or metastatic relapsed cervical cancer (MRCC) and, if possible, during the treatment [57]. They identified 5 significant nonsynonymous mutated genes (*PIK3CA, BRAF, GNA11, FBXW7*, and *CDH1*) in MRCC samples as metastatic recurrence significantly mutated (MSG) genes and found that patients with MRCC who had any detectable MSG mutation had much shorter progression-free survival (PFS) and OS times than those without detectable MSG mutations. In addition, the analysis of matched plasma before and after chemotherapy showed that the decrease in the number of MSG mutations after chemotherapy was closely related to partial remission (PR) and disease stability (SD). In patients with longitudinal tracking of ctDNA analysis, the increase of MSG mutation was observed earlier in response to disease progression than with radiology.

### Clinical application of ctDNA in CIN

In order to eliminate cervical cancer globally, WHO calls for the adoption of innovative technologies to detect cervical precancerous lesions, namely, cervical intraepithelial neoplasia 2–3 (CIN2-3), and the adoption of appropriate strategies to improve the coverage and acceptance rate of cervical cancer screening. Therefore, there is an urgent need for a high-quality screening method to distinguish CIN lesions, which can be cured before they develop into cervical cancer and occur as early as possible. Most studies focus on cervical samples because the cervix is easy to obtain. There are few studies on the detection of circulating diagnostic biomarkers of CIN. Sometimes, they only appear as a control group.

Data on circulating HPV DNA in the blood of CIN patients are limited. Some studies found no HPV DNA in the blood samples of CIN patients. Sathish, N. et al. tested 10 CIN plasma samples of CIN patients, they confirmed the existence of HPV DNA in their tumor tissues but no plasma [58]. Ho, C. M. et al. collected 20 CIN III, 10 CIN II and 10 CIN I blood and cervical swab samples and examined HPV 16, 18 and 52 DNA with qPCR to explore the prevalence of HPV DNA and the amount of virus at diagnosis. However, they found that there was no HPV DNA in the blood of these patients [59]. Kay, P. et al. found no HPV 16 or 18 DNA in the blood of 12 women with precursor cervical lesions with HPV 16 or 18 infections [60]. These results indicate that HPV DNA can be found in the blood of patients with advanced cancer but not in the blood of patients with precursor lesions. Conceicao Gomes Nascimento et al. showed that HPV DNA was identified in the cervix (48.8–55.5%), blood (44.1–77.7%), and matched plasma and cervix samples (32.5–44.4% of CIN patients). The most common types identified in the cervix were HPV16, 18, 31, 33, 58, and 70, whereas HPV16, 18, 33, 58, and 66 were the most common types identified in the plasma [61]. Cocuzza, C. E. et al. reported that 34.2% (41/120) of the patients with a recent record of cervical dysplasia (from CIN2-3 to normal degenerative cytology) were positive for HPV DNA in plasma. HPV detection in cervical and plasma samples rose with the degree of cervical dysplasia, ranging from 15.4 to 38.9% [62]. The researchers summarized that circulating HPV DNA in plasma samples may represent an exciting discriminative biomarker for premalignant conditions, and additional research must confirm their findings.

## Discussion

Although HPV vaccines and cervical cancer screening have shown great positive effects in some developed countries, the incidence and mortality of cervical cancer are still rising worldwide [2, 3]. This shows that the prevention and treatment of cervical cancer are urgent. Universal vaccination and screening are considered effective measures to prevent cervical cancer [4]. The WHO has proposed a range of early screening methods, but most currently focus on cervical samples and suffer from low specificity and sensitivity [63]. Blood-based biomarker screening, on the other hand, can improve this problem. With the development of high technology, the sensitivity and specificity of detecting ctDNA are getting higher and higher. It makes ctDNA suitable as a plasma biomarker for screening cervical cancer and precancerous lesions. Still, several questions remain to be addressed:

### Concordance of HPV sequences in plasma and tissue

Many studies have concluded that HPV sequences obtained from plasma and cervical tissue are 100% consistent [58, 64]. Typing of HPV-DNA positive samples by restriction fragment length polymorphism (RFLP) revealed that HPV DNA could be detected in the cervical tissues in 55 of 58 patients (94.8%) and the plasma in 8 of these patients (11.8%) [58]. The HPV types detected in these eight patients’ cervical tissue and plasma were consistent. However, another study found more HPV genotypes in plasma samples than cervical cancer tissue [65]. In another study, HPV ctDNA was a true reflection of the complete HPV pattern observed in tumors: approximately 95.0% (77/80) of patients with HPV-positive tumor tissue also had positive plasma HPV ctDNA, whereas only 1.9% (1/54) of patients with HPV-negative tumor tissue had positive plasma HPV ctDNA [66]. The issue of HPV DNA concordance in tumors and plasma is yet to be supported by more data and needs to be validated by multicenter studies with large samples.

### The detection rates of HPV DNA

The detection rate of HPV DNA in blood samples from patients with cervical cancer has been reported to be variable in different reports (Table 3). These differences in detection rates may be due to differences in the specimens tested, the method used to extract DNA, the platform used to analyze the DNA, and the primers selected (L1, E6, E7) [59, 60, 64, 67, 68]. Based on the results of the available studies, qPCR, ddPCR, and NGS-based CaptHPV are the most effective detection methods. However, in terms of convenience and price, the common qPCR and ddPCR may be more suitable than others. In particular, ddPCR is a little more expensive than qPCR, but its sensitivity and specificity are better than traditional qPCR, so it is more suitable for ctDNA detection of cervical cancer or precancerous lesions.

### The detection rates of different ctDNA

Although ctDNA from a number of genes has been used as a screening target for cervical cancer, its diagnostic positivity rate varies widely. For example, a study using ddPCR to detect ctDNA in serum found that the HPV *E7* gene was the most sensitive test as it was 70% positive for cervical cancer lesions, which was significantly better than HPV integration sites and *PIK3CA* mutations (39%, 30%); negative HPV *E7* ctDNA in serum after treatment for cervical cancer predicted better PFS for patients; *E7* ctDNA could be detected about 10 months before the clinical diagnosis of recurrence [39]. However, the problem with these studies is mainly that the sample sizes are too small to give convincing conclusions. In addition, there are fewer ctDNA studies for other genes, so it is possible to rapidly develop studies in this area. Therefore, the value of ctDNA for different genes in predicting cervical cancer and precancerous lesions is different, and the possibility of finding more sensitive indicators is crucial for the early screening of cervical cancer or precancerous lesions. Further efforts are warranted to standardize analysis platforms and incorporate ctDNA as a companion biomarker.

### Perspectives and challenges

As a reference tool for tumor treatment and prognosis, ctDNA has some limitations. One is that its short half-life is fewer than 120 min, so its presence can reflect the amount of tumor DNA in real-time, but it needs to be detected by a rapid test method [69]. This requirement for rapid processing also poses a challenge to obtaining samples with less invasive procedures, so optimizing the sampling process from acquisition to processing is still necessary. Moreover, another limitation of ctDNA in cervical cancer is the lack of sensitivity of ctDNA in the detection of early-stage cancers or precancerous lesions [70], as well as the ability of ctDNA to distinguish cervical cancers from other human papillomavirus-associated cancers (e.g., head and neck cancers), which need to be validated in large prospective studies. A further limitation to the use ctDNA in clinical applications is that the ctDNA tests are not cheap and may have some problems in popularizing its use. Therefore, developing cheaper test reagents or assays can promote the clinical use of ctDNA in early cancer screening, cancer treatment, and cancer prognosis.

## Conclusions

In conclusion, ctDNA testing based on blood sampling has the potential to be an effective alternative to traditional tissue sampling methods. It is characterized by simple sample acquisition, high sensitivity, high specificity, and simple and inexpensive assays, so it is potentially clinically useful for the early detection and treatment of cervical cancer and precancerous lesions. To date, small sample sizes have limited most studies evaluating ctDNA technology. Before it can become a standard for routine clinical use, efforts are needed in several directions to remedy the problems: to conduct larger, higher quality studies to provide more rigorous evidence; to find more relevant genes for testing; to assess the consistency of blood and tissue specimens, and to be able to detect precancerous lesions sensitively. However, current evidence suggests that ctDNA may be used as a complementary diagnostic tool rather than a sole decisive biomarker.

## Data Availability

Not applicable.

## Abbreviations

ARMS: Amplification refractory mutation system
cfDNA: Cell-free DNA
CIN: Cervical intraepithelial neoplasia
CRT: Chemoradiotherapy
ctDNA: Circulating tumor DN
ddPCR: Droplet digital PCR
dPCR: Digital PCR
HPV: Human papillomavirus
HR-HPV: High-risk HPV
IARC: International Agency for Research on Cancer
LACC: Locally advanced cervical cancer
LCR: Long control region
LOH: Loss of heterozygosity
LR-HPV: Low-risk HPV
MRCC: Metastatic relapsed cervical cancer
MSG: Metastatic recurrence significantly mutated
NGS: Next generation sequencing
OS: Overall survival
PFS: Progression-free survival
PR: Partial remission
qPCR: Quantitative PCR
SCC-Ag: Squamous cell carcinoma antigen
SD: Disease stability
TAM-Seq: Tagged-amplicon deep sequencing
vh-DNA: Virus-host chimera DNA
